# A Review of Pellets from Different Sources

**DOI:** 10.3390/ma8041413

**Published:** 2015-03-27

**Authors:** Teresa Miranda, Irene Montero, Francisco José Sepúlveda, José Ignacio Arranz, Carmen Victoria Rojas, Sergio Nogales

**Affiliations:** Department of Mechanical Engineering, Energy and Materials, Industrial Engineering School, University of Extremadura, Av. Elvas s/n, 06006 Badajoz, Spain; E-Mails: imontero@unex.es (I.M.); fsepulveda@unex.es (F.J.S.); jiarranz@unex.es (J.I.A.); cvrojas@unex.es (C.V.R.); senogalesd@unex.es (S.N.)

**Keywords:** pellets, characterization, standard, biomass, waste, bulk density, durability, ultimate analysis

## Abstract

The rise in pellet consumption has resulted in a wider variety of materials for pellet manufacture. Thus, pellet industry has started looking for alternative products, such as wastes from agricultural activities, forestry and related industries, along with the combination thereof, obtaining a broad range of these products. In addition, the entry into force of EN ISO 17225 standard makes wood pellet market (among other types) possible for industry and household purposes. Therefore, wastes that are suitable for biomass use have recently increased. In this study, the main characteristics of ten kinds of laboratory-made pellets from different raw materials were analyzed. Thus, we have focused on the most limiting factors of quality standards that determine the suitability for biomass market, depending on the kind of pellet. The results showed considerable differences among the analyzed pellets, exceeding the limits established by the standard in almost all cases, especially concerning ash content and *N* and *S* composition. The requirements of the studied standard, very demanding for certain factors, disable the entry of these densified wastes in greater added value markets.

## 1. Introduction

In January 2014, the European Union presented some climate and energy targets to achieve in 2030 [1], which imply more contribution of renewable energies to the energy mix, although with a postponement of the achievement for these goals. The preceding scenario, the well-known 20-20-20 target, was signed in 2008 and implied, compared to 1990, 20% reduction in CO_2_ emissions by 2020, consuming 20% less energy (with efficient and saving measures) and generating 20% primary energy with renewable sources [2]. This target has been replaced by a new scenario that proposes 27% for renewable contribution to the European energy mix, along with 40% greenhouse gases reduction by 2030.

Among the renewable energies that are available for the achievement of these targets, biomass stands out. It is defined as the biodegradable fraction of the products and wastes of biological origin that come from agricultural activity, forestry and related industries.

Due to the extended use of fossil fuels, biomass was a secondary energy source for decades, with a minimal contribution to the production of primary energy. Currently, and due to several factors, such as the increase of oil prices, the growth in agricultural production and the subsequent need of searching for alternative uses of the generated wastes, biomass has reappeared as an energy source [3,4].

The use of biomass as an energy source provides substantial socio-economic and environmental benefits, compensating its localized nature for its high availability.

However, bio-fuels have low bulk densities which limit their use to areas around their origin; plus, their heterogeneity is considerable when it comes to moisture and granulometry, among others. These drawbacks are restrictive factors for their energy use [5].

Nevertheless, densification minimizes these disadvantages being a process that compress raw materials in order to obtain denser fuels, with homogeneous properties and size. Among the different techniques that are available, pelletizing is currently the most extended one [6].

As a result, global pellet production has considerably increased for the last years. Between 2006 and 2012, pellet production worldwide grew from 7 to 19 million tons [7], with Europe and North America being responsible for, practically, the whole production and consumption of densified products.

The growth in pellet consumption has resulted in more diversity, when it comes to the use of raw materials for pellet manufacture. Consequently, the industry has started looking for products, such as wastes obtained from forestry, agriculture or a combination of the latter, currently obtaining a wide range of these products [8].

This differentiation originated the development of quality standards in some countries, so as to guarantee the right use of the different types of pellets in combustion equipment. In 2011, the implementation of EN 14961 standard [9] regulated densified product market, making the development of manufacture technology possible and encouraging international trade of these products [10]. Recently, EN ISO 17225 standard [11] replaced the former specification and introduced new classifications, such as wood pellets for industrial application, whose demands were lower than the ones corresponding to residential and tertiary use. Consistently, this fact entailed the expansion of the group of wastes suitable for both uses.

Densified products have been widely studied, due to the high availability of different types of biomass, from different origins and with different characteristics, and the need to adapt them to a specific use.

In this way, Miranda *et al.* [12] studied pelletizing of forest wastes from oak forest cleaning. Filbakk *et al.* [13] analyzed the effect of bark content in wild pine pellets by using different proportions of wood and bark, observing better mechanical properties and higher ash percentage in those made from pure bark. Montero *et al.* [14] and Sepúlveda [8] manufactured pellets from different wastes of cork industry, stressing their characteristics and their corresponding densification, with the aim of making a specific use feasible. Serrano *et al.* [15] made barley straw pellets with different moisture and several pine sawdust ratios to improve durability. Mediavilla *et al.* [16] produced pellets by combining vine shoot and cork wastes, optimizing the pelletizing process and reducing total demanded energy. Zamorano *et al.* [17] and García-Maraver *et al.* [18] assessed the influence of the main pelletizing parameters on different wastes from olive pruning. Arranz [5] made different pellets by mixing wastes from Pyrenean oak, olive and grape pomace and cork dust, pointing out that adding olive and grape pomace to Pyrenean oak increased heating value and bulk density of the final product.

In this sense, and based on the abundant literature about this subject, the aim of this work was to analyze the main characteristics of a wide range of laboratory-made pellets from different sources and nature, paying attention to their differences and particularities. Furthermore, the main limiting factors for the compliance with the required standard were assessed, with the purpose of determining the best commercialization for each case, depending on the attributes of the densified product.

## 2. Materials and Methods

EN ISO 17225-1 standard [11] states an initial classification for biofuels, according to their origin and source. Thus, this standard distinguishes different groups: woody, herbaceous, fruit and aquatic biomass, along with their combinations or mixtures. Woody biomass comes from trees, thickets and bushes. Herbaceous biomass is obtained from plants that do not have woody stem and wither at the end of the growing season. Fruit biomass corresponds to plants with seeds. Aquatic biomass is related to plants from aquatic environments. Finally, the terms combinations and mixtures refer to materials from diverse origin, being mixed intentionally (combinations) or not (mixtures).

On this basis, two different kinds of pellets from each group were selected in this work, excluding aquatic biomass, combinations and mixtures, whose use is still at an early stage. On account of the importance of woody biomass, three different sub-groups, with different characteristics, were established: forest wastes, wastes from wood industry and woody agricultural wastes. Table 1 shows the selected pellets.

The data used in the present work were obtained from several publications. Each of them are briefly explained below, pointing out the source, characteristics and pre-treatments that were carried out on raw materials in order to obtain the studied pellets.

▪ Pyrenean oak from an oak grove in the southwest of Spain. All the samples were chipped, ground and dried to 10% moisture (wet basis, wb), and mean values corresponding to different branch sizes were used [12].
▪ Pyrenean sylvestris from a pine grove in Norway. After cutting down, the logs were piled up for two months and then they were chipped. The samples were air dried until moisture was between 11% and 14% (wb). Finally, the samples were ground and sieved [13].
▪ Granulometric separation powder from cork industries in the southwest of Spain. This waste was obtained by grinding the cork sheets that are not suitable for natural cork stoppers and after the selection (according to density) of granulometric separation powder used in manufacture of agglomerate cork stoppers. No pre-treatments were necessary for pelletizing [14].
▪ Pine sawdust from sawmills in the center of Spain. Pre-treatments for pelletizing were not specified [15].
▪ Vine shoots from vineyards located in the north of Spain that were chipped by using a blade mill (10 mm opening width) and, after that, ground by a hammer mill (4-mm mesh size) [16].
▪ Olive branches obtained by pruning in different farming exploitations located in the south of Spain. The samples were chipped at the place of origin and moved to a pelletizing plant, where the samples were ground to reduce their particle size (between 6 and 8 mm). Moisture was reduced below 15% (wb) by a rotating dryer [17].
▪ Barley straw from the Technical and Agronomical Institute of Albacete (Spain) [15]. The samples were ground in a hammer mill and dried to 8% (wb).
▪ Wheat straw from Belgium. No pre-treatments before pelletizing were specified [19].
▪ Olive pomace from an olive mill industry located in the southwest of Spain. This waste, obtained after drying to 10%–15% (wb), did not need further treatments before pelletizing [20].
▪ Grape pomace from a wine industry in the southwest of Spain. The samples were ground by a hammer mill, not needing moisture reduction [5].

**Table 1:** Analyzed pellets.

Biomass Groups		Wastes	Nomenclature
Woody Biomass	forest wastes	pyrenean oak	*PO*
		pyrenean sylvestris	*PS*
	wastes from wood industry	granulometric separation powder from cork industries	*CP*
		pine sawdust	*SW*
	woody agricultural wastes	vine shoots	*VS*
		olive branches	*OB*
Herbaceous Biomass	herbaceous agricultural wastes	barley straw	*BS*
		wheat straw	*WS*
Fruit biomass	agro-industrial wastes	olive pomace	*OP*
		grape pomace	*GP*

The most representative characteristics were selected for the studied pellets. Thus, some properties such as moisture (*M*, % wb), bulk density (*BD*, kg/m^3^ wb), durability (*DU*), chemical composition (*C*, *H*, *N* y *S*, % dry basis, db), ash content (% db) and high heating value (*HHV*) were studied. Low heating value (*LHV*) and energy density (*E*) were calculated from the expressions (1) and (2):

(1) $$LHV=HHV-2.447\;\times \;\left(\frac{H\text{(\%db)}}{100}\right)\;\times \;9.011{\text{ [MJ kg}}^{-1}\text{(db)]}$$

(2) $$E=BD\times LHV{\text{ [MJ m}}^{-3}\text{(wb)]}$$

Finally, the properties of the pellets and the limit values in EN ISO 17225 standard were compared. Even though the standard recommends the origin of the raw material for each specification, in the present work a comparison at all levels was carried out, with the aim of proving the differences among the diverse quality standards. For pellets from woody biomass, Part 2 was used (EN ISO 17225-2) [21], including the following categories: A1, A2 and B, for commercial and household use and I1, I2 and I3 for industrial applications. The remaining pellets were compared with Part 6 (EN ISO 17225-6) [22], concerning pellets from non-woody biomass. For wastes from herbaceous biomass, we followed the specifications for wheat straw, whereas for wastes from agricultural activities the categories A and B were used. Table 2 and Table 3 show the limit values specified in the standard.

**Table 2:** Limit values for pellets from woody biomass.

Property	Commercial and Residential Applications			Industrial Applications		
	A1	A2	B	I1	I2	I3
*M* (% wb)	≤10	≤10	≤10	≤10	≤10	≤10
*BD* (kg/m^3^·wb)	≥600	≥600	≥600	≥600	≥600	≥600
*DU* (%)	≥97.5	≥97.5	≥96.5	≥97.5	≥97.0	≥96.5
*N* (% db)	≤0.3	≤0.5	≤1.0	≤0.3	≤0.3	≤0.6
*S* (% db)	≤0.04	≤0.05	≤0.05	≤0.05	≤0.05	≤0.05
Ash (% db)	≤0.7	≤1.2	≤2.0	≤1.0	≤1.5	≤3.0
*LHV* (MJ/kg·wb)	≥16.5	≥16.5	≥16.5	≥16.5	≥16.5	≥16.5

**Table 3:** Limit values for pellets from non-woody biomass.

Property	Herbaceous Biomass	Fruit Biomass	
		A	B
*M* (% wb)	≤10	≤12	≤15
*BD* (kg/m^3^·wb)	≥600	≥600	≥600
*DU* (%)	≥97.5	≥97.5	≥96.0
*N* (% db)	≤0.7	≤1.5	≤2.0
*S* (% db)	≤0.10	≤0.20	≤0.30
Ash (% db)	≤6.0	≤6.0	≤10
*LHV* (MJ/kg·wb)	NR	≥14.5	≥14.5

NR, not required.

## 3. Results and Discussion

### 3.1. Characterization of Analyzed Pellets

Pellet quality depends both on physical, chemical and mechanical properties of biomass (as a raw material) and pelletizing variables (such as pressure, temperature, *etc.*) [23,24].

Table 4 shows the main characteristics of the analyzed pellets in this study.

Moisture, bulk density and durability are three of the main properties to evaluate densified products.

Moisture must be low in pellets. High moisture levels imply that combustion heat is partially used to evaporate water from the biofuel, whereas for dry biofuels the whole heat is used for the right purpose. Lower moisture contents allow higher flame temperatures (with better temperature gradient and heat transfer, and enabling the completion of combustion), and shorter residence times in the combustion chamber [25,26]. The analyzed pellets showed moisture contents fewer than 10% (wb), except for *VS*, whose value was slightly above this percentage. The results obtained for this parameter were similar, changing depending on the moisture of the raw material, the drying process and operating conditions of the equipment.

**Table 4:** Characteristics of the studied pellets.

Property	PO	PS	CP	SW	VS	OB	BS	WS	OP	GP
*M* (% wb)	6.35	9.50	8.02	9.30	10.80	6.50	7.20	9.40	6.86	7.05
*BD* (kg/m^3^·wb)	678	675	697	650	700	582	644 *	620	780	824
*DU* (%)	95.41	97.20	96.79	98.20	98.80	97.50	95.50	94.40	91.41	85.83
*C* (% db)	50.94	51.80	50.50	50.50	46.90	47.02	43.85	45.10	51.42	42.97
*H* (% db)	6.34	5.70	5.80	6.10	5.70	7.62	5.50	6.00	6.56	9.28
*N* (% db)	1.81	0.37	0.43	0.48	0.58	0.34	0.77	0.91	1.98	2.05
*S* (% db)	0.10	0.03	0.03	0.03	0.05	0.00	0.10	0.00	0.10	0.17
Ash (% db)	2.48	2.50	4.81	0.90	7.10	3.32	10.51	9.10	5.55	7.47
*HHV* (MJ/kg·db)	19.30	19.70	21.41	20.80	18.70	18.82	17.43	18.25	22.03	19.54
*LHV* (MJ/kg·wb)	16.76	16.69	18.52	17.69	15.61	16.03	15.05	13.73	19.17	16.26
*E* (MJ/m^3^·wb)	13,363	11,267	12,907	11,496	10,927	9335	9695	8,510	14,954	13,396

* Bulk density in *BS* samples was obtained from Serrano *et al.* [15].

As far as bulk density is concerned, Mani *et al.* [27] considered that it is the main quality indicator for pellets, along with size distribution, moisture and compression strength during the process. Most of the pellets, except for *OB*, had densities above 600 kg/m^3^ (wb), which is the lower limit demanded by the standard used in this study. Some values were exceptionally high, e.g., for *OP* and *GP* (780 and 824 kg/m^3^ wb, respectively). In general, pellet density decreases in inverse proportion to particle size. This is due to the fact that, during the process, the smallest particles have higher surface areas, obtaining higher density values [6]. In this way, pre-treatments such as grinding or sifting are usually necessary for high-quality pellet manufacture.

The densification ratios, defined as the quotient between the bulk densities of pellets and raw material, changed considerably depending on the density of the raw material, with values between 1.14 for *OP* and 3.32 for *PO*. Thus, lower bulk densities implied higher compaction values.

On the other hand, durability is related to the effectiveness of densification. Pellets should endure different efforts during shipping, loading and transportation to the final destination. Otherwise, considerable amounts of dust might be generated, going into the combustion chamber without burning and increasing emissions and wastes at home, which could imply health risks (explosive atmospheres, respiratory diseases, *etc.*) [28,29]. Most of the pellets had durability values above 95% and slightly less than the lower limits demanded by the standard. These results are common for laboratory-made pellets, where the equipment are smaller and work with less working pressure than the ones used in industries [30]. Pellets from pine sawdust, vine shoots and olive branches would be suitable for a proper transport, handling and further use.

**Figure 1:**
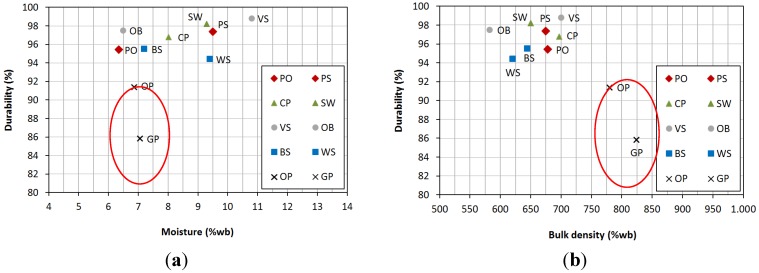
Relationships between (**a**) moisture and durability; (**b**) bulk density and durability.

The following figures show the relationship between the variables that were commented above. Figure 1a shows that the highest durability values were obtained for different moisture levels, between 6.50% and 10.80% (wb), not observing a direct correlation between both variables. Nevertheless, the lowest durability values corresponded to those pellets with very low moisture contents (that is, *OP* and *GP*), which might be due to the defective agglomeration that was observed.

Figure 1b shows the relationship between bulk density and durability. The majority of the pellets, with densities between 582 and 700 kg/m^3^·wb showed high durability values. The highest density values were obtained for agro-industrial wastes (*OP* and *GP*), showing the lowest durability. Therefore, and according to data, very high bulk density does not imply a proper compaction.

The knowledge of ultimate analysis is important to determine the thermal properties of biofuels. Thus, correlations between heating value and carbon, hydrogen and oxygen percentages were observed [31,32].

At the same time, ultimate analysis is useful for predicting the elements that cause an increase in harmful emissions, one of the main problems for the use of mixed biomass [24].

The samples showed different *S* and *N* contents (Table 4). Pellets of *PO*, *BS*, *WS*, *OP and GP* exceeded the upper limit for *N* (0.6% db) that is considered to be acceptable for nitrogen oxide emissions [33,34]. *S* and *N* levels were similar for the pellets belonging to woody biomass, except for *PO*, whose values were extremely high.

**Figure 2:**
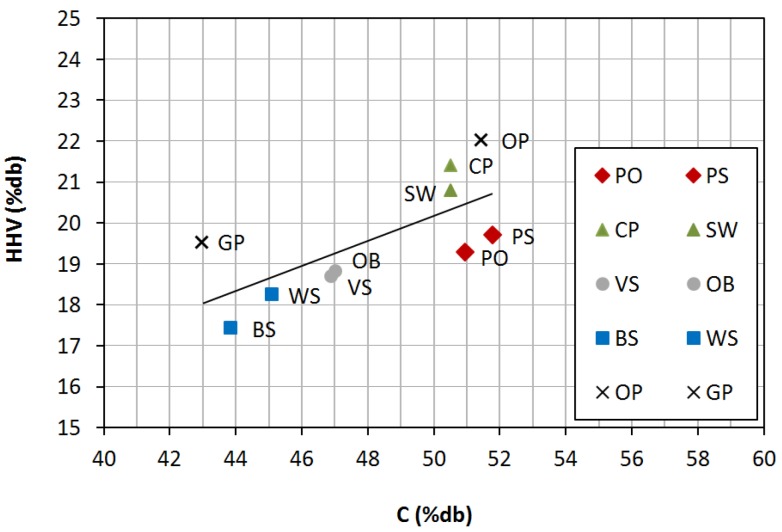
Relationship between carbon content (*C*) and high heating value (*HHV*).

Figure 2 shows the relationship between carbon content and *HHV* for the studied pellets. It can be observed that, the higher *C* percentage was, the higher *HHV* was found, regardless of the raw materials. This is on account of the fact that *C* and *H* are responsible for the energy content in biofuels, due to exothermal reactions that take place with *O_2_* during combustion, generating *CO_2_* and *H_2_O*, respectively [23]. Likewise, *C* and *HHV* values were similar, in each group, for the couple of selected pellets, except for agro-industrial wastes, which is due to the diversity in pellets with the same origin. The highest *HHV* corresponded to forest wastes and wastes from wood industry, along with olive pomace.

Ash content is, by itself, a parameter representative enough for an initial and prompt evaluation of pellets and their quality [7]. Ash contents were diverse. Herbaceous agricultural wastes showed very high levels, as in the case of woody agricultural wastes, with amounts much larger than forest wastes’.

Lehtikangas [29], who studied sawdust pellets, wastes from wood industry and pine cork, and Filbakk *et al.* [13], who also analyzed ash content in pellets from pine wood, bark and their mixtures, concluded that higher bark ratios correspond to higher ash contents. Thus, the lowest ash content was observed for pellets from pine sawdust (0.9% db), possibly on account of stripping the bark from wood before processing.

**Figure 3:**
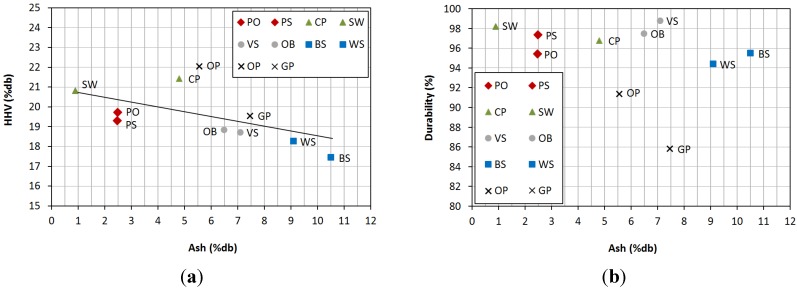
Relationship between (**a**) ash and *HHV*; (**b**) ash and durability.

Figure 3 shows the relationship among ash content, *HHV* and durability. The first relationship is direct: the higher ash content was, the lower *HHV* was found. This fact is in accordance with literature, as published by Monti *et al.* [35], who registered a decrease of 0.2 MJ/kg in *HHV* when ash content increased 1% for six different energy crops, and by Gillespie *et al.* [28], who carried out a study about predicting some quality parameters in pellets according to ultimate analysis data, finding that higher ash levels were related to lower heating values per unit mass of fuel.

The second relationship, between ash content and durability, is not as direct as in the previous case. Generally, high ash content corresponds to low durability. Although Filbakk *et al.* [13] found higher durability values with high fixed carbon percentages, acceptable durability values (according to the standard) were obtained in pellets with higher ash content, as in the case of *CP*, *VS* and *OB*. For woody biomass, bark content increased ash percentage in densified products, but did not reduce its agglomeration capacity.

Energy density is obtained by multiplying bulk density by *LHV* (wb), and quantifies the energy available per time unit. The highest values were obtained for agro-industrial wastes (that is, *OP* and *GP*), due to their high bulk densities and *LHV*, followed by forest wastes, wastes from wood industry and herbaceous agricultural wastes.

**Figure 4:**
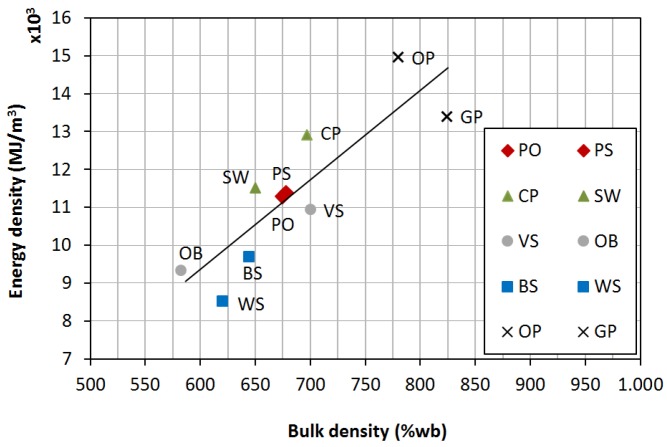
Relationship between bulk density and energy density.

Figure 4 shows the relationship between bulk density and energy density.

Biofuels with high density and high *LHV* have more energy per unit volume. Pelletizing considerably increases energy density, which in turn reduces both logistic and storage needs. For instance, energy density for *CP* increased from 6,873 to 12,907 MJ/m^3^ (wb) [14]. For *OP*, it increased from 11,406 to 14,954 MJ/m^3^ (wb) [20]. Assuming that heating value will be almost the same both in raw materials and their corresponding densified products, energy density increase will be mainly influenced by the equipment and its compaction performance that will determine bulk density in manufactured pellets.

### 3.2. Comparison with Standards

Table 5, Table 6 and Table 7 show the comparison of the studied pellets with the reference standard and its limits (as observed in Table 2 and Table 3). Apart from accepted (OK) and rejected values (not OK), a third result was added to label those properties that do not fit the standard just over (or under) 5% of the upper (or lower) limit, with the aim of pointing out the possible variability due to the inherent limits of the use of laboratory equipment.

According to the results, none of the pellets that were analyzed in this study met all the specifications. *SW* pellets were the ones with better properties, close to the standard requirements, with *N* and ash content slightly higher than the upper limits for the most demanding category (A1).

Overall, the laboratory-made pellets met the requirements concerning moisture and bulk density, except for *VS* and *OB*, respectively. Nevertheless, these problems could be easily solved with a more precise control of the process (pre-drying, more working pressure, *etc.*).

Concerning durability, all the samples, except for *OP* and *GP*, showed high values, above 94%, with the lower limit between 96.0% and 97.5%, depending on the category. These results could be improved by adding additives or other residues with higher lignin content [29] (wood, sawdust, *etc.*), on condition that both have similar ash melting behavior.

**Table 5:** Comparison with EN ISO 17225-2 standard (part 1).

Property	*PO*						*PS*						*CP*					
	A1	A2	B	I1	I2	I3	A1	A2	B	I1	I2	I3	A1	A2	B	I1	I2	I3
*M*	√	√	√	√	√	√	√	√	√	√	√	√	√	√	√	√	√	√
*BD*	√	√	√	√	√	√	√	√	√	√	√	√	√	√	√	√	√	√
*DU*	¡?	¡?	¡?	¡?	¡?	¡?	¡?	¡?	√	¡?	√	√	¡?	¡?	√	¡?	¡?	√
*N*	x	x	x	x	x	x	x	√	√	x	x	√	x	√	√	x	x	√
*S*	x	x	x	x	x	x	√	√	√	√	√	√	√	√	√	√	√	√
Ash	x	x	x	x	x	√	x	x	x	x	x	√	x	x	x	x	x	x
*LHV*	√	√	√	√	√	√	√	√	√	√	√	√	√	√	√	√	√	√

√, OK; x, not OK; ¡?, not OK by less than 5%.

**Table 6:** Comparison with EN ISO 17225-2 standard (part 2).

Property	*SW*						*VS*						*OB*					
	A1	A2	B	I1	I2	I3	A1	A2	B	I1	I2	I3	A1	A2	B	I1	I2	I3
*M*	√	√	√	√	√	√	x	x	x	x	x	x	√	√	√	√	√	√
*BD*	√	√	√	√	√	√	√	√	√	√	√	√	¡?	¡?	¡?	¡?	¡?	¡?
*DU*	√	√	√	√	√	√	√	√	√	√	√	√	¡?	¡?	¡?	¡?	¡?	¡?
*N*	x	√	√	x	x	√	x	x	√	x	x	√	x	√	√	x	x	√
*S*	√	√	√	√	√	√	x	√	√	√	√	√	√	√	√	√	√	√
Ash	x	√	√	√	√	√	x	x	x	x	x	x	x	x	x	x	x	x
*LHV*	√	√	√	√	√	√	x	x	x	x	x	x	¡?	¡?	¡?	¡?	¡?	¡?

√, OK; x, not OK; ¡?, not OK by less than 5%.

**Table 7:** Comparison with EN ISO 17225-6 standard.

Property	*BS*	*WS*	*OP*		*GP*	
			A	B	A	B
*M*	√	√	√	√	√	√
*BD*	√	√	√	√	√	√
*DU*	¡?	¡?	x	¡?	x	x
*N*	x	x	x	√	x	¡?
*S*	√	√	√	√	√	√
Ash	x	x	√	√	x	√
*LHV*	NR	NR	√	√	√	√

NR, not required; √, OK; x, not OK; ¡?, not OK by less than 5%.

Concerning *N* and *S* content, there was a wide range of results. Even though *S* content was kept at around the upper limits, N values exceeded these limits for the most demanding categories, that is, *NO_x_* emissions could be unacceptable. Thus, *NO_x_* emissions depend on nitrogen content in biomass fuels, proportionally increasing with the latter and *O_2_* concentration during combustion [36].

On the other hand, most of the pellets exceeded upper limits for ash content, due to bark (in the case of woody biomass) and impurity content. Although ash accumulation is no longer an unsolvable problem, due to the new automatic systems devoted to ash removal (especially applied to small-scale equipment), it is still an essential condition for residential and tertiary market or industrial use (less demanding than the previous ones).

Concerning heating value, all the pellets, except for *VS* and *OB*, showed values over the upper limits in the standard, recommending the mixture with other products that can improve this property.

Finally, and even though in this work we have included the main properties in order to define pellet quality, it would be necessary the study of further variables such as *Cl* content, whose presence increases corrosion problems, fouling and *HCl* emission, and ash composition and ash melting behavior, that might provoke adherence to heat exchange surfaces (smaller temperature range compared to conventional fuels).

## 4. Conclusions

The most relevant findings in the present work were the following:

▪ The present work points out the possibility of using different kinds of pellets for different purposes, due to the EN ISO 17725 entry into force. This fact makes it possible, in turn, an increase in biomass contribution to the energy mix, contributing to the achievement of EU’s objectives.
▪ For the studied pellets, the properties were highly scattered, even for those pellets that share the same group, as a consequence of the different origins and natures, the use (or not) of pre-treatments and the different degrees of control over manufacture.
▪ Higher *C* percentages corresponded to higher *HHV*, and these parameters were similar for each couple of pellets from the same group, regardless their origin. Equally, higher bulk densities corresponded to low-durability pellets. Therefore, high bulk density did not imply proper agglomerations.
▪ The different results obtained for the pine wastes showed the importance of pre-treatments in order to make the most of biomass by-products. Thus, stripping the bark from the wood is essential when it comes to woody biomass densification.
▪ Concerning the standard that was used in this study, moisture, density and durability demands were generally met by the samples, not being a problem in manufacture real conditions. Ash content was, by far, the most demanding parameter, being exceeded by most of the laboratory-made pellets.
▪ Regarding marketing destination, only pine sawdust pellets (without bark) could access domestic sector that usually demands high-quality products with minimum ash content. The remaining woody biomass pellets, except for vine shoots, could be used in industry and tertiary sector, depending on the characteristics of combustion equipment.
▪ Pellets from non-woody biomass, especially fruit biomass, compared favorably with many parameters of the standard, although the specifications are less demanding than in the case of woody biomass.
▪ Even though EN ISO 17225 standard enables the commercialization of pellets from different raw materials for different purposes and applications, the specifications to be considered are very demanding and, in certain cases, unattainable for some kinds of biomass, limiting their use to specific equipment, designed for this purpose.

## Glossary

Symbol	Concept	Units
*db*	Dry basis	% db
*wb*	Wet basis	% wb
*BD*	Bulk density	kg/m^3^·wb
*DU*	Durability	%
*E*	Energy density	MJ/m^3^
*HHV*	High heating value	MJ/kg
*LHV*	Low heating value	MJ/kg
*M*	Moisture	% wb
*BS*	Barley straw	-
*CP*	Granulometric separation powder from cork industries	-
*GP*	Grape pomace	-
*OB*	Olive branches	-
*OP*	Olive pomace	-
*SW*	Pine sawdust	-
*PO*	Pyrenean oak	-
*PS*	Pyrenean sylvestris	-
*VS*	Vine shoots	-
*WS*	Wheat straw	-
